# Functional Polymorphisms in* IRAKs* Are Related to Hepatocellular Carcinoma Risk in Chinese Population

**DOI:** 10.1155/2018/1252849

**Published:** 2018-07-09

**Authors:** Hui Wang, Ci Song, Qi Qi, Tongtong Huang, Lijuan Wang, Jianguo Chen, Jian Zhu, Zhibin Hu, Juncheng Dai

**Affiliations:** ^1^Department of Epidemiology and Biostatistics, School of Public Health, Nanjing Medical University, Nanjing 211166, China; ^2^Jiangsu Key Lab of Cancer Biomarkers, Prevention and Treatment, Collaborative Innovation Center For Cancer Personalized Medicine, Nanjing Medical University, Nanjing 211166, China; ^3^Qidong Liver Cancer Institute, The First People's Hospital of Qidong, Qidong 226200, China

## Abstract

**Background:**

Interleukin 1 receptor associated kinases* (IRAKs)* play a central role in TLR signaling pathway. Scarce literature has investigated the association of potential functional genetic variants of* IRAKs* with Hepatitis B Virus- (HBV-) related hepatocellular carcinoma (HCC).

**Methods:**

A case-control study with 1,538 HBV-positive HCC patients and 1,465 chronic HBV carriers was conducted to evaluate the effects of common missense variants of IRAK family members on HCC. Proliferation assays and real-time polymerase chain reactions were carried out to evaluate the functions. Multivariable adjusted logistic regression was adopted to estimate effect size and identify risk factors.

**Results:**

Association analysis indicated that rs4251545 A allele of* IRAK4* (p.Ala428Thr) was positively associated with HBV-related HCC risk (OR = 1.30, 95% CI: 1.09–1.54, *P* = 0.003). Functional annotation indicated that rs4251545 reduced its own expression in liver (*P* = 0.031). Further molecular functional analysis detected that rs4251545 increased the proliferation rate of L02 cells (*P* < 0.05). Meanwhile, rs4251545 reduced mRNA expressions of IL-6, IL-8, CXCL-1, and CXCL-2 in L02 cells (*P* < 0.01).

**Conclusion:**

rs4251545 of IRAK4 (p.Ala428Thr) modified the susceptibility to HBV-related HCC via increased proliferation rate and reduced production of inflammatory cytokines and chemokines. Further well-designed experiments are warranted to validate our findings.

## 1. Introduction

Globally, HCC was the second leading cause of cancer-related death in men [1]. The incidence rate of HCC varied greatly over the world. East, Southeast Asia, and Sub-Saharan Africa were most vulnerable to HCC, which was consistent with the high prevalence of HBV infection in these regions. China was one of these typical countries and accounted for almost half part of HCC incidence worldwide [1]. The main risk factors of HCC were chronic infections with the hepatitis B or C viruses and/or intake of contamination foods with aflatoxin B1. In particular, hepatitis B virus (HBV) infection was the key risk factor in the pathogenesis of HCC in China [2, 3]. However, it was known that around 1.3%–14.9% HCC was caused by chronic HBV carrier, especially for those who had cancer family history subjects [4, 5].

Toll-like receptors (TLRs) were one of the conserved and universally expressed innate immunity family members and detected various microbe-associated molecular patterns as the first line of defense [6, 7]. Upon the activation of TLRs, interleukin-1 receptor associated kinase 4 (IRAK4) and IRAK2 were sequentially recruited to the myeloid differentiation primary response gene (MyD88) via its death domain, which further formed a structure called Myddosome [8, 9]. Finally, the NF-*κ*B or IRF was activated and released the proinflammatory cytokines, such as IL-6 and IL-8, and interferons [10]. IRAK1 was phosphorylated by IRAK4 and activated the downstream signaling, while IRAK3 was considered as an inhibitor of TLR signaling. However, the precise function of each of them was not very well known.

To date, several documents illustrated that genetic variants of TLRs were involved in the pathogenesis of HCC [11–13]. However, only one research found that IRAK2 (p.Leu392Val) carriers had attenuated auto clearance rate of hepatitis C virus (HCV) [14]. Limited systematic researches were performed to evaluate the potential functional variants of IRAKs in the pathogenesis of HCC at population scale, particularly for HBV-related HCC. Therefore, the present study was conducted to explore the association between potential functional single nucleotide polymorphisms (SNPs) of IRAKs and HBV-related HCC with a case-control design (1,538 HBV-positive HCC patients and 1,465 chronic HBV carriers) in Chinese population. Functional assays were further carried out to dissect the molecular mechanisms.

## 2. Material and Methods

### 2.1. Subjects and Variants Selection

#### 2.1.1. Subjects

Participants were all Chinese Han, including 1,538 HBV-positive HCC patients and 1,465 HBV-positive controls. Detailed information about baseline characteristics and ethical review were published in the previous study [15].

#### 2.1.2. Variants Selection Strategy and Functional Annotation

*IRAKs *related potential functional variants were systematically selected according to the following criteria: (i) minor allele frequency (MAF) at least 5% in Chinese population (phase II + III Feb 09, on NCBI B36 assembly, dbSNP b126 HapMap SNP database); (ii) *P*-value of Hardy-Weinberg equilibrium (HWE) test in controls >0.05; (iii) autosome related; (iv) potential functional annotation, such as missense variants. Finally, 3 SNPs (rs3844283 of* IRAK2 *(p.Leu392Val), rs1152888 of IRAK3 (p.Ile86Val), rs4251545 of IRAK4 (p.Ala428Thr)) were selected for further evaluation. Potential functional annotation was performed in Genotype-Tissue Expression (GTEx, v6) database (https://www.gtexportal.org/home/testyourown).

#### 2.1.3. Genotyping Methods

Variants selected in our study were derived from our previous HCC GWAS dataset, which was imputed based on Illumina Human omniExpress 12v1 chips (imputed by IMPUTE2 and the haplotype information from the 1000 Genomes Project and HapMap 3 [15]).

### 2.2. Molecular and Functional Evaluation

#### 2.2.1. Cell Lines

HepG2 cells were cultured in Minimal Essential Medium (MEM; Hyclone). Human hepatoblastoma HepG2.2.15 cells (Shanghai Yu Bo Biotech Co., Ltd) and Human liver normal cells L02 were cultured in Dulbecco's Modified Eagle Media (DMEM). All cells were supplemented with 10% fetal bovine serum (Sigma) and 1% Penicillin-Streptomycin (Beyotime) and cultured at 37°C with 5% CO_2_.

#### 2.2.2. Plasmids, Cloning, and Site-Directed Mutagenesis

Gateway-compatible entry clone of human* IRAK4 *was a kind gift from Prof. Alexander N. R. Weber (Tuebingen University, Germany). Mutations in IRAK4 were introduced into IRAK4 WT using a Quikchange XL kit (Agilent). PCR conditions were followed the instruction of the kit. Sequences of mutagenesis primers were designed by Agilent and available upon request. Detailed information of site direct mutagenesis was presented in Supplementary Document 1. Finally, the mutation sites were confirmed by the DNA sequencing (Genscript Ltd, Nanjing, China).

#### 2.2.3. Cell Proliferation Assay

Proliferation rate of transfected cells was measured by the Cell Counting Kit-8 system (CCK8, Dojindo Laboratory, Japan) according to the manufacturer's instructions. Briefly, cells were collected after 24 h transfection with 100 ng plasmids of interest genes and resuspended and reseeded into 96 well plates at 6000 per well for HepG2 and HepG2.2.15 and 3000 per well for L02. The absorbance was detected by adding 10 ul of CCK8 solution into each well for 2 hours and measured at 450 nm at 12 h, 24 h, 36 h, 48 h, and 72 h, individually.

#### 2.2.4. RNA Isolation and Quantitative Real-Time PCR Assay

Total RNA extracted from cells with the TRIzol LS Reagent (Invitrogen, Carlsbad, CA, USA) according to the manufacturer's instruction. The concentration and quality of the RNA were measured by NanoDrop 2000 (Thermo Fisher Scientific, Waltham, MA, USA), and 500 ng RNA was reverse transcribed to cDNA using the Prime-script RT Master Mix (TaKaRa, Kyoto, Japan) in accordance with the manufacturer's instructions. The expression levels of IL-8, IL-6, CXCL-1, and CXCL-2 were detected by using SYBR green labeling kit (TaKaRa, Kyoto, Japan) and performed in ABI 7900 real-time PCR system (Applied Biosystems, Foster city, CA, USA). Each sample was prepared in triplicate, and experiments were done by three independent assays. The results of relative mRNA expression levels were calculated via the equation 2^−ΔCt^, in which ΔCt = Ct_gene_ − Ct_GAPDH_, and further normalized to expression level of empty vector. The sequences of primers were provided in Supplementary Table 1.

### 2.3. Statistical Analysis

The associations between genotypes and HBV-related HCC risks were estimated in an additive model by logistic regression, adjusting for age, sex, smoking, and drinking status. Results were presented as odds ratios (ORs) and 95% confidence intervals (CIs). Homogeneity test among different strata according to selected variables was assessed with *χ*^2^-based *Q* test. The proliferation and mRNA expression differences were detected by Student's *t*-test (equal variance assumed). All of the statistical analyses were performed in Stata 11.0 (Stata Corporation, College Station, Texas, USA), and statistical significance was set at two-sided *P* ≤ 0.05.

## 3. Results

### 3.1. rs4251545 (IRAK4 p.Ala428Thr) Was Associated with HBV-Related HCC Risk

**Figure 1:**
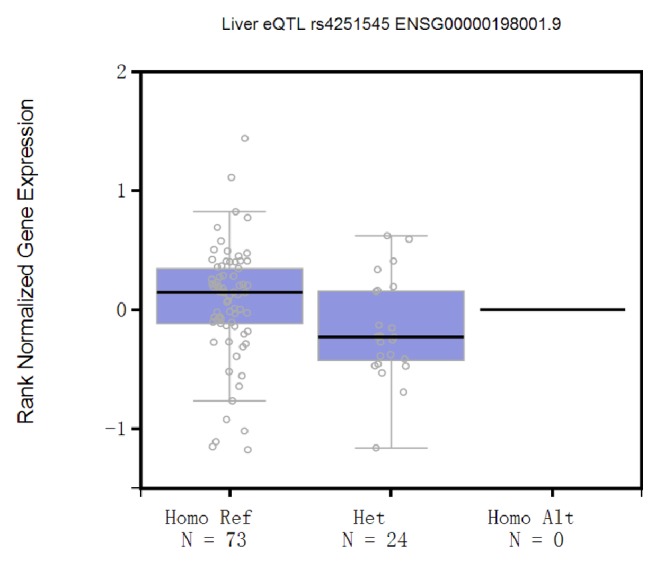
*IRAK4 expression level in liver*. Data were obtained from GTEx analysis release V6p (dbGaP Accession phs000424.v6.p1), *P* = 0.031. The black line in the box indicated the mean value of expression level of each genotype. The upper hinge and lower hinge indicated the maximum and minimum expression level of each genotype. Each dot represented one specific genotype carrier.

The association analysis indicated that only 1 coding variant, rs4251545 A allele (IRAK4, p.Ala428Thr), significantly increased the risk of HCC [(OR = 1.30 (1.09–1.54), *P* = 0.003, Table 1)]. However, no significant association was observed between the rest two variants (rs3844283 and rs1152888) with HCC risk. Further subgroup associations between rs4251545 and the risk of HCC were analyzed in subsamples according to various age, sex, smoking status, and drinking status. No significant heterogeneity was observed among different strata after stratification analyses (Table 2). Meanwhile, the geographic localization might impact on the pathogenesis of HBV-related HCC; the stratification analysis was performed as well. The *P* value was statistically significant in the Southern China [Guangdong, 1.37 (1.13–1.66)] and this difference was attenuated in the Eastern China population (Shanghai). Overall, the whole subjects did not present heterogeneity in terms of geographic locations (Supplementary Table 2). Additionally, the result of potential functional annotation from GTEx indicated that only rs4251545 (IRAK4, p.Ala428Thr) reduced its own expression level in liver tissue (*P* = 0.031, Figure 1). Hence, further analysis focused on rs4251545.

### 3.2. rs4251545 (IRAK4 p.Ala428Thr) Increased the Proliferation Rate in L02 Cells

**Figure 2:**
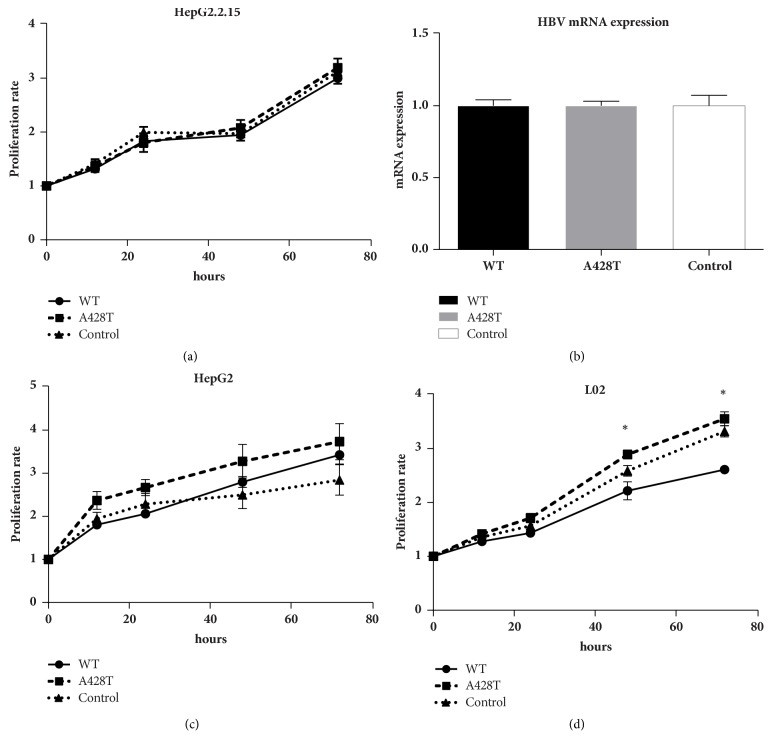
*The proliferation rate of rs4251545 (IRAK4 p.Ala428Thr) among different liver cell lines. *(a) 100 ng plasmid of IRAK4 428Ala or IRAK4 428Thr (rs4251545) was transfected into HepG2.2.15 cells, respectively, then the proliferation rate was measured at 12 h, 24 h, 36 h, 48 h, and 72 h, individually. Empty vector was set as the negative control. The dot, square, and triangle referred to control, IRAK4 428Ala, and IRAK4 428Thr, respectively. (b) mRNA of HBV was detected after transfection of IRAK4 428Ala or 428Thr in HepG2.2.15 cells, individually. (c) and (d) were the plasmids of interest transfect into HepG2 cells (c) and L02 cells (d), then the proliferation was measured at 12 h, 24 h, 36 h, 48 h, and 72 h, individually. Empty vector was set as the negative control. The dot, square, and triangle referred to IRAK4 428Ala (WT), IRAK4 428Thr (A428T), and control, respectively. *∗* represented that the comparison between IRAK4 428Ala and IRAK4 428Thr was statistically significant by Student's *t*-test, at *P* < 0.05.

To further explore the biological function of rs4251545 in liver cancer, we conducted the proliferation assays in different liver cell lines. The IRAK4 428Ala or IRAK4 428Thr was transiently transfected into HepG2.2.15 cells, HepG2 cells, and L02 cells, respectively. The proliferation rate of IRAK4 428Thr was significantly enhanced in L02 cells (*P* < 0.05). However, no statistical difference was observed in HepG2.2.15 and HepG2 cells (Figure 2). Moreover, the mRNA level of HBV was not impacted when comparing the overexpressed IRAK4 428Ala and IRAK4 428Thr into HepG2.2.15 cells (Figure 2).

### 3.3. rs4251545 (IRAK4 p.Ala428Thr) Attenuated mRNA Expression Levels of* IL-6*,* IL-8*,* Cxcl-1*, and* Cxcl-2*

**Figure 3:**
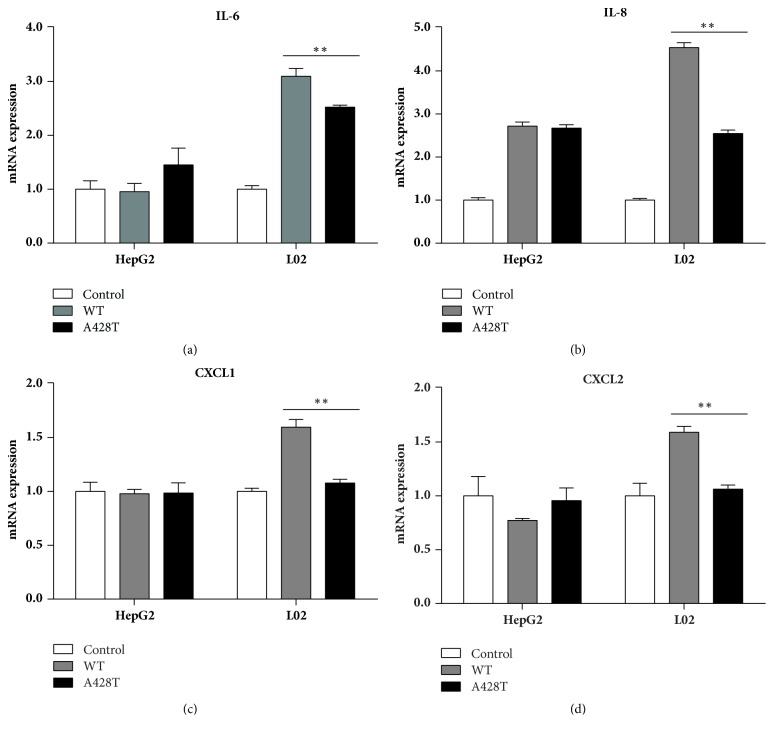
*The mRNA expression of selected proinflammatory and chemokines in HepG2 and L02 cells*. 100 ng plasmid of IRAK4 428Ala and IRAK4 428Thr was transfected into HepG2 and L02 cells, respectively, then the mRNA expression was detected for IL-6 (a), IL-8 (b), CXCL-1 (c), and CXCL-2 (d) after 48 h. Empty vector was set as the negative control. *∗∗* represented that the comparison between IRAK4 WT and IRAK4 A428T was statistically significant by Student's *t*-test, at *P* < 0.01.

After transfection of IRAK4 428Ala or IRAK4 428Thr 48 h, the mRNA expressions of inflammatory cytokines were assessed by the RT-PCR, such as* IL-6*,* IL-8*,* CXCL-1*, and* CXCL-2, *respectively. All results indicated that IRAK4 428Thr reduced the expression of inflammatory cytokines and chemokines in L02 cells (all *P* < 0.01), while no significant impact was detected in HepG2 cells (Figure 3).

## 4. Discussion

Present study used a case-control design and found an unfavorable association between rs4251545 (IRAK4 p.Ala428Thr) and HBV-related HCC even after adjusting basic social demographic factors, which could be attributed to the diminished signaling activation of NF-*κ*B (IL-6 and IL-8) and chemokines (CXCL-1 and CXCL-2). These results indicated that IRAK4 should play an important role in the pathogenesis of HCC, which could be set as a target for the future individual treatment.

Our results were consistent with a previous study which found that rs4251545 was associated with 1.68- to 4.99-fold increases in the risk of developing breast cancer in African-American women [16]. Moreover, rs4251545 locus occurred in an enhancer/silencer region of the gene, which might impact IRAK4 transcriptional level. This conclusion was consistent with the result from GTEx, which indicated that rs4251545 did reduce the expression level of IRAK4 per se and consequentially reduced the expression of proinflammatory cytokines.

The plausibility of reduced proinflammatory and increased proliferation rate of cells could be attributed to the unbalanced immunity reaction. Several studies found that dysregulation of TLR signaling pathway activation might shift the balance between the production of pro- and anti-inflammatory cytokines and would have a profound effect on the risk of infection, chronic inflammation, and cancer [2]. Our previous data found that rs3844283 (IRAK2, p.L392V) was associated with reduced NF-*κ*B signaling and IFN-*α* and correlated with reduced HCV auto clearance rate and increased risk of chronic HCV infection [14]. Another study indicated that TLR2-196 to -174 del allele carriers had significantly higher HCV viral loads and lower IL-8 induction, using stimulation of monocytes from TLR2-196 to -174 ins/ins homozygous patients [11]. Interestingly, this study pointed out that the distribution of TLR2-196 to -174 del/ins alleles in HCV infected patients without liver cancer and healthy control was identical. They claimed that the polymorphism did not seem to affect the susceptibility of acquirement of HCV infection; it was more likely to have impact on the prognosis of HCV infection. In our analysis, both cases and controls were infected with HBV. We found that the rs4251545 seems to affect the process and outcome of HBV infection. Additionally, the reduced production proinflammatory cytokines and chemokines and increased proliferation rate were only significant in L02 cells, which meant that the rs4251545 of IRAK4 might involve in the initial step of carcinogenesis. When cells were already malignant, the function might be not as dominant as the initial step.

Several limitations in present study should be mentioned: first, several relevant factors that might partially explain the function of immunity at different stage of carcinogenesis, such as cirrhosis status and viral load, were not included into the analysis due to data availability. Second, external population-based validation study should further solidify our results. Third, the population only restricted to Chinese population which would limit the ability of extrapolation. Finally, further comprehensive functional analyses, such as transwell assay, ELISA assay coimmunoprecipitation, or EMSA, should be carried out to deepen our understanding of the molecular mechanism of the pathogenesis in HCC. Overall, it is the first attempt to evaluate the potential functional variants of IRAKs in the pathogenesis of HCC with a moderate sample size. Our study further confirmed that IRAK4 is involved in progression of HBV-related HCC. However, more well-designed population validation and molecular assays were warranted in the future analysis.

## Figures and Tables

**Table 1:** Association between three SNPs and HBV-related HCC susceptibility.

Gene	SNP	Major/minor	Case^a^ (1538)	Control^a^ (1465)	HapMap^b^ MAF	Case MAF	Control MAF	OR (95% CI)^c^	*P* ^c^	*P*_FDR_^d^
*IRAK2*	rs3844283	C/G	1178/344/16	1086/349/30	0.19	0.12	0.14	0.87 (0.74–1.02)	0.082	0.085
*IRAK3*	rs1152888	A/G	261/731/546	252/722/491	0.38	0.41	0.42	0.94 (0.84–1.04)	0.243	0.243
***IRAK4***	**rs4251545**	**G/A**	**1198/309/31**	**1203/250/12**	**0.07**	**0.12**	**0.09**	**1.30 (1.09**–**1.54)**	**0.003**	**0.003**

Note: MAF = minor allele frequency. ^a^Major homozygote/heterozygote/rare homozygote between case and control subjects. ^b^HapMap (phase II + III Feb 09, on NCBI B36 assembly, dbSNP b126, HapMap-CHB). ^c^OR (95% CI) and *P *are derived from logistic regression analysis after adjusting for age, sex, drinking and smoking status. ^d^*P* value for multiple comparison, using B-H FDR (false discovery rate) method.

**Table 2:** Stratified analyses of association between rs4251545 and HCC risk.

Variables	rs4251545			
	Case^a^	Control^a^	OR (95% CI)	*P* ^b^
Age				0.746
<60	962/247/27	967/206/10	1.30 (1.08–1.57)	
≥60	236/62/4	236/44/2	1.38 (0.92–2.07)	
Gender				0.774
Male	1061/274/28	934/191/10	1.32 (1.09–1.59)	
Female	137/35/3	269/59/2	1.26 (0.82–1.93)	
Smoking				0.760
Never	712/182/20	547/110/6	1.28 (1.01–1.64)	
Ever	486/127/11	656/140/6	1.31 (1.03–1.66)	
Drinking				0.545
Never	784/202/23	702/156/8	1.23 (0.99–1.53)	
Ever	414/107/8	501/94/4	1.46 (1.10–1.93)	

^a^Major homozygote/heterozygote/rare homozygote between case and control subjects. ^b^Homogeneity test among different strata according to selected variables was assessed with *χ*^2^-based *Q* test.

## Data Availability

The data used to support the findings of this study are available from the corresponding author upon request.

## Abbreviations

HCC: Hepatocellular carcinoma
HCV: Hepatitis C virus
HBV: Hepatitis B virus
HWE: Hardy-Weinberg equilibrium
IRAKs: Interleukin 1 receptor associated kinases
LD: Linkage disequilibrium
MAF: Minor allele frequency
NAMD: Nanoscale molecular dynamics
ORs: Odds ratios
WT: Wild type.

## Supplementary Materials

Supplementary Materials (1) Supplementary Document 1: the protocol of site direct mutagenesis. (2) Supplementary Table 1: primers of real time polymerase chain reaction. (3) Supplementary Table 2: stratified analyses of association between rs4251545 and HCC risk by geographic localization.
