# Supplementary material for: Functional Polymorphisms in IRAKs Are Related to Hepatocellular Carcinoma Risk in Chinese Population
Source: Biomed Res Int. 2018 Jul 9;2018:1252849. doi: 10.1155/2018/1252849 (PMC6077667; doi:10.1155/2018/1252849)
Supplement: Supplementary Materials — (1) Supplementary Document 1: the protocol of site direct mutagenesis. (2) Supplementary Table 1: primers of real time polymerase chain reaction. (3) Supplementary Table 2: stratified analyses of association between rs4251545 and HCC risk by geographic localization. [file 1252849.f1.pdf]

1. Supplementary Document 1

1). Flowchart of primer design:
Website of Agilent (https://www.agilent.com/) ⟹ Products ⟹ Mutagenesis & Cloning ⟹ Site directed mutagenesis ⟹ Quickchange Lighting ⟹ Free access to Primer Design program ⟹ Paste the FASTA-formatted DNA sequence of target protein in the pointed region (point 3) ⟹ load it ⟹ indicated the nucleotide that you want to change to which one ⟹ specify the position of target nucleotide and click the button named “primer design”, below listed the

Primer Name	Primer Sequence (5' to 3')
Forward	5'-actagcaacagagtacatagtttcaactgaagtggaatcag-3'
Reverse	5'-ctgattccacttcagttgaaactatgtactctgttgctagt-3'

For the mutant strand synthesis reaction (thermal cycling)
2). Prepare the sample reaction(s) as indicated below (Agilent product number #210519)

2.5μl of 10×reaction buffer
*X*μl (50ng) of dsDNA template
*X*μl (50ng) of oligonucleotide primer (forward one)
*X*μl (50ng) of oligonucleotide primer (reverse one)
1μl dNTP mix
0.75 ul of QuikSolution reagent
ddH2O to a final volume of 25μl

Then add:
1μl of QuikChange Lightning Enzyme

3). The cycling parameters outlined as below: (copied from the manuscript of QuikChange Lightning Site-Directed Mutagenesis Kit)

Segment	Cycles	Temperature	Time
1	1	95℃	2 minutes
2	18	95℃	20 seconds
		60℃	10 seconds
		68℃	30 seconds/kb of plasmid length
3	1	68℃	5 minutes

4). *Dpn* I Digestion of the Amplification Products (copied from the manuscript of QuikChange Lightning Site-Directed Mutagenesis Kit)

1). Add 2 μl of the provided *Dpn* I restriction enzyme directly to each amplification reaction. (Notes Use only the Dpn I enzyme provided; do not substitute with an enzyme from another source.)

2). Gently and thoroughly mix each reaction mixture by pipetting the solution up and down several times. Briefly spin down the reaction mixtures and then immediately incubate at 37 ℃ for 5 minutes to digest the parental (i.e., the nonmutated) supercoiled dsDNA.

5). Transformation of XL10-Gold Ultracompetent Cells ((copied and modified from the manuscript of QuikChange Lightning Site-Directed Mutagenesis Kit)

1). Gently thaw the XL10-Gold ultracompetent cells on ice. For each control and sample reaction to be transformed, aliquot 45 μl of the ultracompetent cells to a prechilled 14-ml BD Falcon polypropylene round-bottom tube.

2). Add 2 μl of the β-ME mix provided with the kit to the 45 μl of cells. (Using an alternative source of β-ME may reduce transformation efficiency.)

3). Swirl the contents of the tube gently. Incubate the cells on ice for 2 minutes

4). Transfer 2 μl of the Dpn I-treated DNA from each control and sample reaction to separate aliquots of the ultracompetent cells.

As an optional control, verify the transformation efficiency of the XL10-Gold ultracompetent cells by adding 1 μl of 0.01 ng/μl pUC18 control plasmid (dilute the control provided 1:10 in high-quality water) to another 45-μl aliquot of cells.

5). Swirl the transformation reactions gently to mix and incubate the reactions on ice for 30 minutes.

6). Preheat NZY+ broth in a 42 ℃ water bath for use in step 9.

7). Heat-pulse the tubes in a 42 ℃ water bath for 30 seconds. The duration of the heat pulse is critical for obtaining the highest efficiencies. Do not exceed 42 ℃.

8). Incubate the tubes on ice for 2 minutes.

9). Add 0.5 ml of preheated (42 ℃) NZY+ broth to each tube, then incubate the tubes at 37 ℃ for 1 hour with shaking at 225–250 rpm.

10). Plate the all of transformation reaction solution on the agar plate containing the appropriate antibiotic for the plasmid vector.

11). Incubate the transformation plates at 37 ℃ for >16 hours.

2. Supplementary Table 1 Primers of real time polymerase chain reaction.

Genes	Primers
IL-6	CAGCCACTCACCTCTTCA (F)
	CACTGTCTTTGAGCCTGTC (R)
IL-8	ATGACTTCCAAGCTGGCCGTG (F)
	TTATGAATTCTCAGCCCTCTTCA (R)
Cxcl-1	CGCTCAGTCAGTGAGTCTCTT (F)
	GGGGGACTTCACGTTCACA(R)
Cxcl-2	GGTGCTCAGGAAAGCTGACT (F)
	CAAGCCTAGAGGTCCTTGCC (R)
HBV S Region	TCGTGTTACAGGCGGGGTTT (F)
	GACTGCGAATTTTGGCCAAG (R)
GAPDH	TTCACCACCATGGAGAAGGC (F)
	GGCATGGACTGTGGTCATGAG (R)

3. Supplementary Table 2 Stratified analyses of association between rs4251545 and HCC risk by geographic localization

Variables	rs4251545			
	Case [a]	Control [a]	OR(95%CI)c	Pb
Region				0.488
Shanghai	390/86/4	408/72/4	1.12(0.80-1.56)	
Guangdong	808/223/27	795/178/8	1.37(1.13-1.66)	

[a] Major homozygote/heterozygote/rare homozygote between case and control subjects.

[b] Homogeneity test among different strata according to selected variables was assessed with χ^2 -based Q test.

[c] OR (95% CI) and P are derived from logistic regression analysis after adjusting for age, sex, drinking and smoking status.
